# A Prospective Study on the Impact of the Communication Skills Module on Medication Adherence: Healing Through Dialogue

**DOI:** 10.7759/cureus.97315

**Published:** 2025-11-20

**Authors:** Neena Bhatti, Girish Joseph, Pallavi Abhilasha, Dinesh K Badyal, Mohammad Waseem Faraz Ansari, Aroma Oberoi, Ajay Kumar

**Affiliations:** 1 Department of Pharmacology, Christian Medical College and Hospital, Ludhiana, IND; 2 Department of Psychiatry, Christian Medical College and Hospital, Ludhiana, IND; 3 Department of Community Medicine, Employees State Insurance Corporation (ESIC) Medical College and Hospital, Chennai, IND; 4 Department of Microbiology, Christian Medical College and Hospital, Ludhiana, IND; 5 Department of Biochemistry, Christian Medical College and Hospital, Ludhiana, IND

**Keywords:** 2nd-year mbbs students, adherence, communication modules, competency-based medical education (cbme), prescribed medication

## Abstract

Background: Effective communication is vital for building doctor-patient relationships, improving patient compliance, and reducing medical errors. However, communication training is often underemphasized in medical education.

Objectives: This study aimed to enhance communication skills among second-year MBBS students by developing and validating communication skills modules focused on medication adherence, training faculty to deliver them, implementing the modules, and evaluating their effectiveness.

Methods: A prospective interventional study was conducted in the Department of Pharmacology at Christian Medical College and Hospital, Ludhiana. Two competency-based communication modules were developed and validated in the context of antitubercular drugs and oral contraceptive education. Following ethical approval, the modules were implemented with 101 second-year MBBS students. Faculty members were trained, and students were assessed using a simulated, observed structured practical examination (OSPE). Focus group discussions (n = 8) and faculty interviews (n = 4) were conducted, and all qualitative data were analyzed using the NVivo software (Lumivero, Denver, Colorado).

Results: Postmodule OSPE performance improved in 87.12% of students (p = 0.0002). Student self-assessment results showed improvement, with 78% reporting poor communication before the intervention and 94% reporting significant improvement and increased confidence afterward. Qualitative feedback affirmed enhanced communication competence and awareness of medication adherence and patient safety.

Conclusion: Structured communication modules significantly improved communication skills, confidence, and simulated interactions among second-year MBBS students. Both students and faculty recognized the critical role of communication in patient safety and medication adherence, underscoring the need to integrate such training into medical curricula.

## Introduction

The National Medical Council (NMC) introduced the new competency-based medical education (CBME) curriculum in August 2019. It includes a foundation course, attitude, ethics, and communication (AETCOM), competency-based assessment module, alignment integration module, skill training module, logbook guidelines, and elective modules. The change was not easy for the faculty to accept, as it required a great deal of unlearning. The primary objective of the new curriculum is to produce a competitive Indian medical graduate (IMG) capable of delivering better healthcare outcomes and being globally accepted. The NMC envisions the IMG as a clinician, leader, communicator, lifelong learner, and thorough professional [1].

A doctor-patient relationship (DPR) is vital to medical practice and consultation. Without a good rapport, DPR leads to negative perceptions of the healthcare system, poor medical outcomes, and even violence against doctors [2]. Good communication is the key to a meaningful DPR. Effective communication, a crucial component of medical education, significantly improves patient compliance and overall health outcomes. Strong communicators are better able to understand patients’ needs and address their concerns, which ultimately leads to more effective management of illness [3]. Communication is defined as “a process of exchanging information using verbal and nonverbal methods between two persons: one giving the information and the other at the receiving end.” It also emphasizes the importance of communicating negative or difficult messages without creating conflict or destroying trust [4]. Communication is an essential component of medical education and is defined as “a lifelong learning process stretching from undergraduate to postgraduate and specialty training, and beyond” [5].

Some believe that communication skills cannot be taught to medical students [6]. Although communication skills training is part of the curriculum, it is often perceived as less stimulating by students. A study by Mandal et al. concluded that communication skills can be learned with intentional effort and repetition. Faculty development programs were found to be essential components of teaching-learning activities related to communication training [4]. On the other hand, numerous complications occur due to miscommunication in Indian healthcare settings, leading to allegations and litigation against doctors. Such issues can often be resolved through effective communication between doctors and patients or their relatives [7]. However, there are several hurdles to effective communication, such as excessive use of medical jargon, arrogance on the part of either doctor or patient, language differences, diverse ethnic backgrounds, overburdened clinicians due to high patient load, and frequent interruptions, all of which contribute to noncompliance and drug errors [8-10].

A study reported that while a considerable number of patients had adequate information about their prescribed drugs, many were unaware of warnings, adverse drug reactions, and follow-ups. Another study showed that physician interpersonal skills and teaching had a strong impact on patient adherence, recall, and satisfaction [11]. Training programs should therefore begin early in the undergraduate medical curriculum and be integrated with diverse teaching strategies [4]. This study was conducted to introduce a communication skills module on medication adherence among second-year MBBS students, with the goal of improving compliance, reducing drug errors, and ultimately enhancing patient safety. Although communication is key to ensuring medication adherence and patient safety, structured training in this area is often lacking in undergraduate medical education, particularly in the preclinical phase. This gap highlights the need for early integration of communication modules that link pharmacological knowledge with real-world patient counseling skills. By doing so, we aim to develop competent IMGs who can strengthen the healthcare system by reducing drug errors, improving compliance, and minimizing litigation against young doctors.

## Materials and methods

Population

This study was conducted in the Department of Pharmacology at Christian Medical College and Hospital, Ludhiana. The participants were second-year MBBS students (n = 101) from the 2022 batch at the same institution. All students enrolled in the second-year professional course were invited to participate, constituting a purposive sampling of the study population.

Inclusion Criteria

All second-year MBBS students enrolled in the 2022 batch were included in the study. Students actively participated in the communication skills module, which is part of the curriculum.

Exclusion Criteria

No formal exclusion criteria were applied, as participation in the communication skills module was a mandatory component of the curriculum, and a waiver of consent was granted by the Institutional Ethics Committee (IEC).

Intervention

A structured communication skills module was introduced and implemented, focusing on two pharmacology competencies. The study focused on two competencies from the pharmacology communication skills curriculum: 1) PH 5.2 (Communicating with patients about the optimal use of drug therapy, devices, and storage of medicines): This competency emphasizes the importance of clear and effective communication to ensure that patients understand how to take medications correctly, manage devices appropriately, and store medicines safely. Developing proficiency in this area helps reduce medication errors and enhances patient adherence; and 2) PH 5.3 (Motivating patients with chronic diseases to adhere to prescribed management): This competency targets the ability to counsel and encourage patients, particularly those with chronic conditions, to follow their treatment plans consistently. It focuses on motivational strategies, addressing barriers to adherence, and fostering patient engagement in their own care.

The intervention included teaching-learning sessions, faculty sensitization, and student assessment through an observed structured practical examination (OSPE) and feedback.

Comparison

Pretest scores and student perceptions before module implementation served as the baseline for comparison with posttest scores and feedback obtained after the module. No separate control group was used.

Outcomes

The quantitative outcomes include improvement in student knowledge, attitudes, and skills related to medication adherence and communication, assessed through pre- and posttests using the OSPE checklist (based on the Kalamazoo model). The qualitative outcomes include insights from student focus group discussions (FGDs) and faculty interviews regarding module effectiveness, relevance, and perceived impact on learning. The overall goal is to enhance communication competence, promote medication adherence, reduce drug errors, and improve awareness of patient safety.

Study design

A core committee was established to develop and implement the communication skills module. The committee consisted of four pharmacologists and one member from the clinical department, ensuring a multidisciplinary perspective in the module’s design. Prior to initiating the study, ethical clearance was obtained from the IEC of Christian Medical College and Hospital, Ludhiana (approval number: IECBHMR/202409-452). Following ethical approval, the core committee participated in a series of sensitization and brainstorming sessions. These sessions were aimed at aligning the team on the study objectives, identifying key competencies to be addressed, reviewing existing teaching-learning strategies, and discussing innovative methods for integrating communication skills training into the pharmacology curriculum. The collaborative approach ensured that the module was both evidence-based and tailored to the educational needs of second-year MBBS students.

The modules covered drug therapies and competencies related to antitubercular (anti-TB) drugs and oral contraceptive pills (OCPs). A needs assessment was conducted among the pharmacology faculty. The faculty members voluntarily participated to identify existing gaps, perceptions, and training needs related to teaching communication skills in pharmacology. A structured questionnaire, developed by the core committee and validated by members of the Medical Education Unit (MEU), non-MEU members, and five students from the 2021 MBBS batch, was used. It included both closed-ended (Likert-scale) and open-ended questions.

Following the implementation of the two modules, FGDs were conducted with eight students. The students were assigned alphabet letters to maintain confidentiality. Academic members from outside the pharmacology department assisted in documenting the qualitative data collected from the FGDs. Additionally, semistructured, in-depth interviews were conducted with four faculty members who had been trained and had experience using the modules. The doctors were assigned numbers to maintain confidentiality. These interviews were audio-recorded. All qualitative data from the FGDs and interviews were analyzed using NVivo software (Lumivero, Denver, Colorado).

Statistical analysis

The results obtained were in percentages and proportions. Qualitative data from FGDs and faculty interviews were analyzed using thematic analysis with NVivo software, version 14. Quantitative pre- and posttest data were compared using a paired t-test to assess changes in students’ knowledge, attitudes, and communication skills.

Evaluation

Evaluation was done for all the stakeholders, including faculty and students.

Needs Assessment Questionnaire

A structured questionnaire developed by the core committee, validated by the MEU members, non-MEU members, and five students from the 2021 MBBS batch, was used. It included both closed-ended (Likert-scale) and open-ended questions.

Student Evaluation

Students were assessed using an OSPE checklist immediately after module implementation. The checklist consisted of questions derived from the Kalamazoo scale, which are presented in Appendices 1 and 2. The purpose of the evaluation was to assess changes in knowledge and attitudinal aspects related to communication about drug use (anti-TB/OCP) and patient motivation.

Feedback Questionnaire

A structured feedback questionnaire was administered to students after the implementation of the module to obtain their perceptions regarding its effectiveness. The questionnaire primarily focused on postimplementation evaluation, assessing the relevance, clarity, and overall impact of the module on their learning experience. This is provided in Appendix 3.

Interviews

Interviews were conducted only for the four faculty members. The format for the interviews was semistructured. The purpose was to explore their experiences in delivering the modules, challenges faced, and perceptions of student engagement. The interviews were recorded and transcribed.

FGD

It was conducted with eight students using a structured questionnaire. The purpose was to obtain qualitative insights into students’ learning experiences, perceived relevance, and suggestions for improvement.

## Results

This study involved 101 students and four faculty members to examine the importance of communication skills. A needs assessment questionnaire was completed by the faculty, which revealed that all faculty members perceived an emphasis on communication skills to be equally important as technical knowledge for patient safety, contributing to a holistic approach that integrates emotional support and education with medical care. They also highlighted the role of communication in empowering patients, encouraging participation in healthcare decisions, and managing difficult interactions, cultural differences, and professionalism in diverse settings.

An OSPE assessment conducted pre- and postmodule implementation showed that 87.12% of students had improvement after the module implementation. A p-value of 0.0002 indicates that there is a significant difference between pre- and postimplementation of the module. The details are shown in Table 1.

**Table 1:** Improvement in students after module implementation Data are presented as mean ± SD. Statistical significance calculated using a paired t-test. The total number of students is 101 SD: standard deviation

Parameter		Value
Preintervention score	Mean ± SD	61.65 ± 10.00
	95% confidence interval	59.70-63.60
Postintervention score	Mean ± SD	64.93 ± 10.38
	95% confidence interval	62.91-66.95
Improvement in pretest score (%)		87.12
No improvement in pretest score (%)		12.88
p value		0.0002

Almost all faculty members perceived that the students were not confident and were unable to convey their message to the simulated patient during the OSPE before the module implementation. About 75% of the faculty felt that communication skills should be taught separately rather than as part of AETCOM. The faculty expressed a neutral opinion regarding the student’s interaction with the simulated patient as a person, with empathy, beyond their disease and demography.

An OSPE assessment pre- and postmodule implementation showed that 87.12% of students had improvement after the module implementation. The students perceived that before the module, 78% of them had inadequate communication skills and struggled with engaging patients, conveying medical information, and showing empathy. After completing the module, 94% reported significant improvements in their communication abilities and felt more confident in their patient interactions.

**Figure 1:**
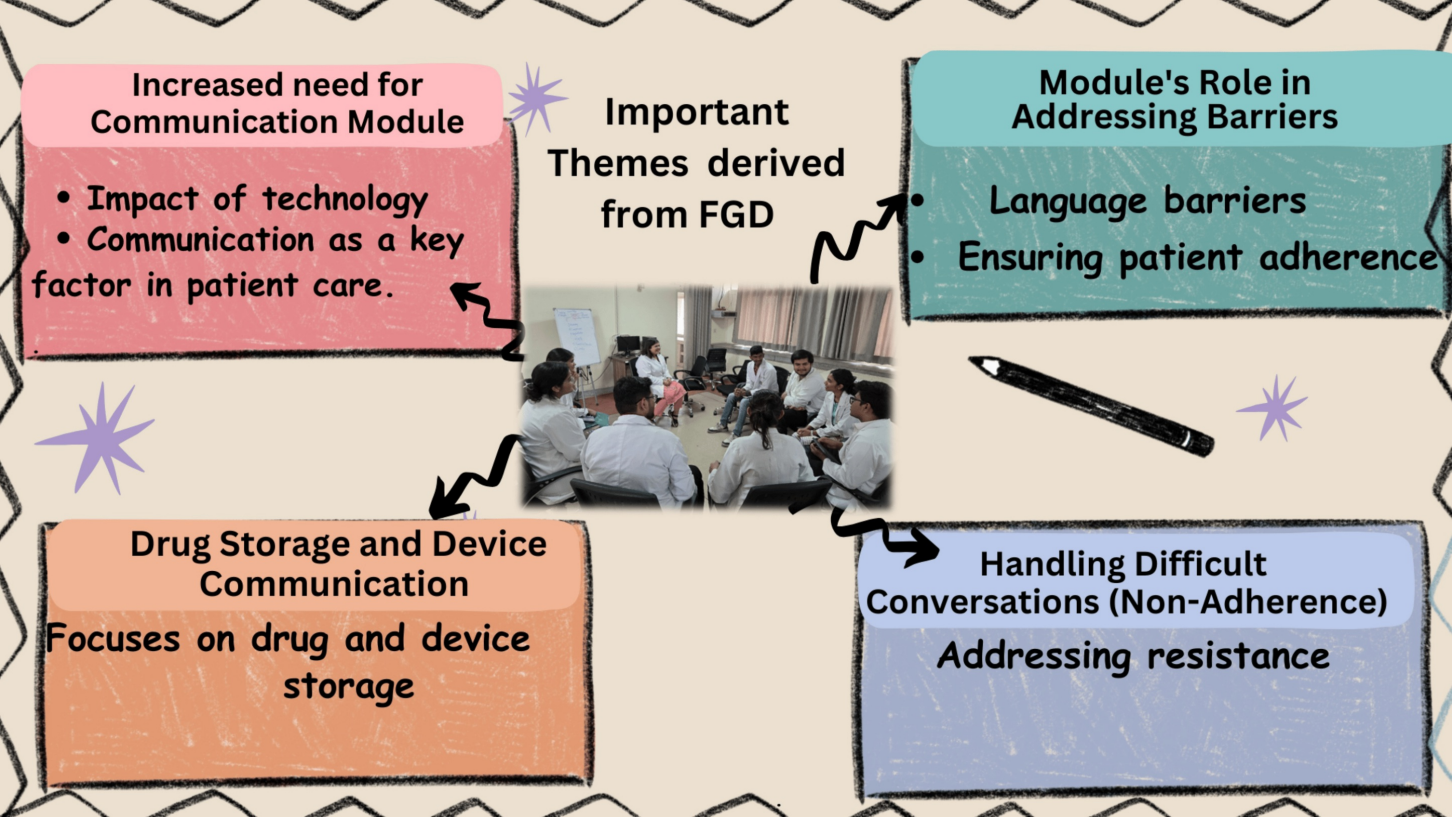
Themes of FGD conducted for students FGD: focus group discussion Image credit: This is an original image created by the author Neena Bhatti

All students agreed that communication skills are essential not only for professional success but also for life in general. They recognized that effective communication is crucial for building connections, conveying information, and promoting positive interactions in both personal and professional contexts. An FGD was conducted with eight volunteer students. Figure 1 shows the themes that were discussed during the FGD.

The students also suggested that doctors should address cultural and societal taboos and discuss the financial aspects of treatment options. The students clearly understood the importance of adherence to prescribed medication, especially in chronic diseases and prolonged medication, as in the case of OCP intake and tuberculosis.

In-depth semistructured interviews were conducted with the faculty who participated in the role play and were part of the module. One of the faculty members pointed out that it is not sufficient to merely provide patients with the “what,” the dosage and frequency of their medication. It is equally important to explain the “why” behind it. Recognizing the high demands on pharmacists’ time, the doctor expressed that it is the responsibility of physicians to ensure that patients fully comprehend the significance of their treatment. Moreover, the doctor stressed that effective communication should be integrated throughout the healthcare system, rather than being treated as an afterthought. A summary of the thematic analysis conducted under various themes is given in Table 2.

**Table 2:** Summary of faculty interviews

Theme	Summary
Theme 1: The importance of communication	Doctors consistently emphasized the vital role of communication skills in patient care, teamwork, and overall healthcare quality
Theme 2: Integrating communication skills into the curriculum	There was a strong consensus on the need for structured communication skills training in medical education, ideally starting early in the curriculum
Theme 3: Potential benefits of a communication skills curriculum	Doctors highlighted numerous benefits of communication skills training, including improved patient understanding, increased adherence to treatment, enhanced empathy, reduced conflicts, and better teamwork
Theme 4: The relationship between communication skills and interpersonal relationships	It enhances patient adherence, reduces errors, lowers anxiety, and boosts satisfaction. Strong communication also supports professional development, reduces litigation risks, and contributes to better overall healthcare quality
Theme 5: Barriers to curriculum delivery	Several barriers were identified, including a lack of faculty time and training, student disinterest, and challenges in translating theoretical knowledge into practical skills
Theme 6: Student and patient barriers affecting treatment	Doctors may neglect to inform patients about potential side effects, like orange-colored urine, leading to panic and treatment discontinuation. Incomplete communication results in poor compliance and treatment failure, with patients often changing doctors. Medical students may struggle to apply communication skills in practice, despite learning them in theory, and patients often express dissatisfaction with poor communication from doctors
Theme 7: Conflict management through communication	Communication was recognized as a crucial tool for managing and resolving conflicts with patients and their families
Theme 8: Additional suggestions	Faculty offered various suggestions for enhancing communication skills development, such as role-playing, mentor shadowing, real patient interactions, and diverse learning methods

The themes were developed iteratively, with codes derived from transcripts of FGDs and faculty interviews. Data saturation was considered achieved when no new codes or themes emerged from additional interviews or discussions, indicating that the collected qualitative data adequately captured the range of participants’ experiences and perceptions regarding the communication skills module.

Overall, the data suggest a strong recognition among faculty the importance of communication skills in healthcare and a need for greater emphasis on communication skills training in medical education. Addressing the identified barriers and implementing the suggested strategies could significantly enhance communication competence among future healthcare professionals, ultimately leading to improved patient care and healthcare outcomes.

## Discussion

This study demonstrates that structured communication skills training significantly improves second-year MBBS students’ ability to convey medical information, engage patients empathetically, and promote medication adherence. Quantitatively, 87.12% of students showed improvement in OSPE scores postmodule implementation (p = 0.0002), reflecting enhanced confidence and competence in patient interactions. Qualitative findings from FGDs and faculty interviews highlighted the perceived importance of communication skills for patient safety, adherence, empathy, conflict management, and professional development. Both students and faculty emphasized the need for the early integration of structured communication training into the curriculum, separate from existing AETCOM components, to address real-world challenges such as cultural barriers, financial discussions, and patient education.

A study conducted in the West revealed that when doctors begin their preregistration training, they typically learn how to prescribe by gradually accumulating bits of knowledge, eventually building their own understanding and skill set. However, when interviews were conducted with doctors who had made significant prescribing errors (most commonly related to incorrect dosages), many admitted that no one had formally taught them about proper dosing [12]. Nearly all students in the present study recognized the importance of acquiring communication skills during medical school, noting that effective communication enhances patient satisfaction by fostering trust, rapport, and better health outcomes.

This study’s findings are comparable to a content analysis that showed gaps in spontaneous prescription discussions, with key information such as adverse drug reactions often missing. In that study, a module based on parts of the PJ-STArT-Block was used. After a brief guided peer discussion and module implementation, the prescription talks became more informative. The written test results revealed that students who conducted or observed the talks performed better than those who did not participate in the module [13]. Before participating in the communication skills module, 78% of the students in our study perceived their abilities as inadequate. They expressed uncertainty in effectively engaging with patients, conveying medical information clearly, and demonstrating empathy. However, after completing the module, 94% reported significant improvement, demonstrating enhanced confidence in their ability to communicate effectively. This aligns with previous findings, where 96.43% of students reported improved communication with patients after receiving structured training [14]. Similarly, another study noted a 78.46% improvement, further supporting the importance of communication skills training in medical education [3].

Other studies have also shown a positive impact of structured communication training on student competence. For example, one report demonstrated increased confidence in interviewing skills among students who received structured training [15]. These results emphasize the importance of integrating communication skills into medical curricula to better prepare students for patient interactions. Despite these positive outcomes, some studies have revealed a gap between student engagement and faculty support. For instance, although the Kalamazoo communication framework was integrated into the MBBS curriculum, students showed low engagement, indicating a need for more relevant and motivating teaching methods. Faculty members strongly supported communication training, recognizing its role in enhancing professionalism and patient interactions. They emphasized that communication skills are as important as understanding technical aspects of medicine, such as drug dosage, frequency, and storage. However, traditional didactic teaching approaches were less effective in engaging students, highlighting the need for innovative and interactive methods [16].

In our study, role play was the primary teaching-learning method used by the pharmacology faculty. A similar study demonstrated that an integrated approach to developing communication skills was well received by both teachers and students. Many students found the interactive sessions and role-playing activities to be the most engaging parts of the training [17]. Additionally, students recognized the importance of clear communication in patient education. In the present study, 97% of students agreed that explaining medication details, including dosage and duration, improves patient compliance. They understood that patients are more likely to adhere to treatment plans when they clearly comprehend the purpose and administration of their medications. This finding reinforces the value of communication skills in patient safety and healthcare outcomes. Moreover, 79% of students acknowledged the importance of giving clear instructions about drug storage, and 95% emphasized the need to confirm patient understanding by asking them to repeat the instructions. This approach effectively reduces misunderstandings and ensures patient safety.

Students in the FGDs cited multiple references related to patient nonadherence. They suggested that explaining resistance, highlighting the seriousness of conditions, and discussing common side effects with patients and families would be more beneficial to patients. Nonadherence is frequently a result of inadequate communication between doctors and patients [18]. Studies have shown that in only about one-third of prescription discussions did physicians address risks and adverse events [19,20], even though these issues are particularly important from the patients’ perspective [21]. Poor communication in this regard is one of the factors associated with poor medication adherence [22]. This underscores the potential significance of prescription discussions in improving adherence. Another study highlighted the need to emphasize this issue in undergraduate medical education [23,24]. A review suggested that using simulated patients holds promise, especially in pharmacological education, as it not only enhances students’ sense of responsibility regarding drug safety but also promotes patient-centered communication [25].

Faculty unanimously agreed on the importance of ensuring patient understanding, highlighting communication as the key to patient education and empowerment. They emphasized its role in fostering autonomy, encouraging active participation, and improving health outcomes. This underscores the value of communication in a holistic approach to patient care. One study demonstrated that a simulation-based pharmacology training module effectively identified students’ deficiencies in prescription discussions and enhanced their performance. The approach not only improved students’ skills during the module but also showed lasting benefits [13]. This study emphasizes the importance of fostering communication skills early in medical education. Integrating structured communication training throughout the undergraduate years helps students refine these skills, enhancing both patient care and professional competence. The positive student attitudes observed highlight the need for such training in the curriculum [26].

The findings of this study align with previous research that supports the effectiveness of structured communication skills training in medical education. Two similar studies reported significant improvements in communication skills among medical students who received structured training. Structured communication training has also been observed to increase students’ confidence and preparedness in clinical settings, as noted by clinical preceptors. These consistent findings emphasize the value of communication skills training in enhancing students’ clinical interactions and overall competence [3,14,15].

Limitations of the study

This study was conducted at a single institution with second-year MBBS students, limiting generalizability. A comparison with unassessed students was not performed, preventing a definitive attribution of improvements solely to the module. Additionally, assessments were immediate postmodule OSPE scores, without long-term follow-up or objective measurement of patient outcomes. Future studies with control groups, longitudinal evaluation, and patient-centered metrics are warranted. If the module could have been repeated for the same group of students, the impact would likely have been deeper. Greater involvement of faculty and alignment with other clinical departments could have made the sessions even more engaging.

Implications of the study

From a policy perspective, integrating structured communication skills training into the undergraduate medical curriculum aligns with national and institutional goals for CBME, patient safety initiatives, and quality improvement frameworks. Widespread adoption of such modules could standardize communication competencies across medical schools, supporting regulatory requirements and promoting the development of healthcare professionals who are better equipped to provide patient-centered care.

Directions for future research

One faculty member suggested introducing such modules in early clinical exposure for first-year medical students, which may have a greater impact if students understand the implications at an early stage. Future efforts should also focus on repeating and adapting this module for other drug therapies and subsequent batches of students.

## Conclusions

Students reported a significant improvement in their communication abilities, confidence, and interactions within a simulated setting after completing the communication skills module. Both students and faculty recognized the vital role of communication in patient safety, treatment adherence, and improving healthcare delivery. The study highlights the need for communication modules that focus on medication adherence and proper drug storage. Despite challenges like limited faculty time and student disengagement, practical approaches such as role-playing and mentor shadowing can further enhance these skills. Strong communication promotes better patient relationships, reduces errors, and improves satisfaction, leading to better healthcare outcomes and professional growth.

## Appendices

Appendix 1

This appendix consists of a checklist for tuberculosis used for assessing the communication skills of students.

**Table 3:** Tuberculosis checklist ADR: adverse drug reaction

Tuberculosis checklist	Marks
Introduce yourself to the patient and open the discussion	1
Gather information regarding his/her lifestyle and hygienic conditions: Always cover your mouth while coughing and sneezing. Wash your hands after coughing or sneezing. Wear a mask when in crowded areas. Stay in isolation at your home as far as possible. Well-ventilated house Well-balanced diet	6
Shares information - Pharmacological measures: A) Take all medications as prescribed. B) Don’t stop treatment yourself. C) Regular follow up every month. D) Sputum smear examination to be done at end of two months. E) ADRs to be informed	5
4. Reach agreement on problems and plans	1
Closure - ensure whether patient understands and have any queries, if any	2
TOTAL	15

Appendix 2

The oral contraceptive pill checklist is shown.

**Table 4:** OCP checklist for communication OCP: oral contraceptive pill

OCP checklist	MM
1.Introduce yourself to the patient and open the discussion	2
2. Gather the information regarding her lifestyle, food habits	2
3. Give information about no. of tablets: 21contraceptive drug +7 iron pills in sequence, start from 5^th^ day of menstrual cycle, one pill at bed time, Effective after 3 months, so additional barrier method to be used until then	3
4. Explain about the missed pill and what to do in case of a missed pill	2
5. Inform the patient about withdrawal bleeding. Explain the ADRs	2
6. Instruct the patient to start a new pack at the end without break	2
7. Closure - ensure whether patient understands and has any queries, if any.	2
TOTAL	15

Appendix 3

Questionnaire for students

Your gender:*

    Female

    Male

1. Do you think your communication skills “BEFORE” the module was satisfactory.*

    Strongly disagree

    Disagree

    Neutral

    Agree

    Strongly agree

2. Do you think your communication skills “AFTER” the module was satisfactory*

    Strongly disagree

    Disagree

    Neutral

    Agree

    Strongly agree

3. To be a successful doctor, strong communication skills one are as important as expanding my medical knowledge.

    Strongly disagree

    Disagree

    Neutral

    Agree

    Strongly agree

4. It’s hard for me to admit that I struggle with communication.

    Strongly disagree

    Disagree

    Neutral

    Agree

    Strongly agree

5. I don’t have the time to invest in learning communication skills.

    Strongly disagree

    Disagree

    Neutral

    Agree

    Strongly agree

6. I find communication skills training engaging

    Strongly disagree

    Disagree

    Neutral

    Agree

    Strongly agree

7. I’m not motivated to attend communication skills sessions.

    Strongly disagree

    Disagree

    Neutral

    Agree

    Strongly agree

8. Developing communication skills has helped improve my teamwork abilities.

    Strongly disagree

    Disagree

    Neutral

    Agree

    Strongly agree

9. Learning communication skills has helped me show greater respect toward patients.

    Strongly disagree

    Disagree

    Neutral

    Agree

    Strongly agree

10. Explaining the drug, dosage and duration of drug to my patient will help to have a better patient compliance.

    Strongly disagree

    Disagree

    Neutral

    Agree

    Strongly agree

11. Instructions regarding the storage of the drugs might not be important.

    Strongly disagree

    Disagree

    Neutral

    Agree

    Strongly agree

12. It's crucial to verify the patient's understanding by asking them to repeat the given instructions.

    Strongly disagree

    Disagree

    Neutral

    Agree

    Strongly agree

13. Patient satisfaction will be more with a good communication.

    Strongly disagree

    Disagree

    Neutral

    Agree

    Strongly agree

14. I have difficulty trusting communication skills lessons delivered by non-clinical faculty.

    Strongly disagree

    Disagree

    Neutral

    Agree

    Strongly agree

15. Learning communication skills has helped me understand patients' rights around confidentiality and informed consent.

    Strongly disagree

    Disagree

    Neutral

    Agree

    Strongly agree

16. Gaining communication skills during medical school is incredibly valuable.

    Strongly disagree

    Disagree

    Neutral

    Agree

    Strongly agree

17. Passing exams will matter more in medical school than mastering communication skills.

    Strongly disagree

    Disagree

    Neutral

    Agree

    Strongly agree

18. Communication skills are vital because they’re essential throughout life.

    Strongly disagree

    Disagree

    Neutral

    Agree

    Strongly agree

19. Would you want this type of module to be a part of your curriculum?*

    Yes

    No
